# Role of MicroRNA-326 and its Target Genes Bcl-xL and Bak as Potential Markers in Platelet Storage Lesion in Blood Banks

**DOI:** 10.1007/s12288-022-01542-0

**Published:** 2022-06-02

**Authors:** Wessam Elgendy, Rania Swelem, Noha Aboudiba, Reham Abo Elwafa

**Affiliations:** Clinical Pathology Department, Faculty of Medicine, Alexandria University, Alexandria, Egypt

**Keywords:** Platelet concentrates, MiRNA, Apoptosis, Bcl-xL, Bak, MiRNA-326, Platelets storage lesion

## Abstract

Platelet transfusion is crucial in the management of various conditions such as quantitative and qualitative platelet disorders. A serious problem that impacts public health is the shortage of Platelet concentrates (PCs) that frequently affect few blood donors’ countries, such as Egypt. This has necessitated the need to establish novel standards for determining the quality of PC during storage. It was found that microRNAs (miRNA) differential expression profile is a helpful tool for recognition of physiological platelet changes during storage. The aim of the current study was to highlight the role of platelet miRNA-326 and its putative target apoptotic genes, Bcl-xL and Bak, and their role in platelet storage lesion (PSL). Differential expression of miRNA-326 and its target genes in the apoptotic pathway, Bcl-xL and Bak was done using quantitative real time PCR (QR-PCR) on different storage points at day 0, day 3 and day 5 in blood bank. The results of the current study revealed over expression of miRNA-326 throughout days of storage resulted in down regulation of Bcl-xL gene and subsequently up regulation of Bak gene. MiRNA-326 contributes to platelet apoptosis and PSL through inhibition of anti-apoptotic Bcl-xL expression and enhancing pro-apoptotic Bak expression. Differential miRNA-326 and its target gene, Bcl-xL and Bak, expression levels at different points of platelets storage are promising tools as biomarkers for platelets undergoing PSL in blood banks.

## Introduction

Each year millions of blood products are transfused, and many lives depend on transfusion [1]. Platelet transfusions are used to control bleeding in patients with quantitative or qualitative platelet dysfunctions [2]. Recently, there was a steady rise on the platelets requirements for transfusion [3].

Platelet products available for transfusion are either random donor (RDP) or single-donor platelets apheresis (SDP).Platelet concentrate (PC) derived from whole blood donations can be either platelet rich plasma (PRP) or buffy coats from multiple donors, using different sequential centrifugation procedures [4].

A major problem that affects public health is the lack of availability of PCs that mainly impact countries with few blood donors, such as Egypt [5, 6]. Also, the possibility of bacterial contamination and PSL; which leads to discarding of all unused PC bags takes place after day 5. All these causes contribute to the missing of PCs from the blood bank stock on the 6th day, leading to major problem in stock management in blood banks. Yet, many of these bags may still have functional platelets [5, 7].

Platelets are anucleated cells, upon storage in blood banks the platelets show morphological and physiological changes called ‘platelet storage lesion’. The PSL seriously affects the structure, function and lifespan of stored platelets; causing them to be ineffective after transfusion [1, 8, 9].

For many decades, platelets were neglected in genomic research as it were thought to function by pre-synthesized and inherited proteins. Although platelets are anucleated cells produced from megakaryocytes cytoplasmic fragmentation, platelets contain various types of RNAs, varying from protein coding messenger RNAs to small non-coding RNAs most of inherited from megakaryocytes. Many necessary components for gene regulation in mature platelets are preserved from megakaryocytes (e.g., pre-miRNAs, Dicer, TRBP2 and Argonaut). [5, 10, 11]

MiRNAs are highly conserved non-protein-coding RNAs. They are small RNA molecules, about 22 nucleotides in their mature state. MiRNAs regulate gene expression in the cells through binding to 3' untranslated region (UTR) of a messenger RNA resulting in its degradation or inhibition of translation according to the degree of complementarity. By this way miRNAs disrupt translation and prevent gene expression. [1, 8]

Apoptosis is defined as programmed process of cell death. Apoptosis plays an important role in PSL upon platelets storage in blood banks [8]. Apoptosis is under control of two main pathways, intrinsic (or mitochondrial) and extrinsic pathways. Platelet life span is under tight control of BCL-2 family, which balances the inhibition or initiation of apoptosis. BCL- 2 family members are divided into three categories: the pro-apoptotic BH3-only proteins, the pore-forming Bax and Bak proteins; and the pro-survival Bcl-2-like proteins (Bcl-2, Bcl-xL, Bcl-w, A1, and Bcl-b) [12].

Human platelets express approximately 750 miRNAs regulating platelet genesis, functions and gene expression [8, 10, 13, 14]. Recent studies revealed that miRNAs play an important role in platelets apoptosis and life span; which makes them be a potential biomarkers for platelet reactivity [8, 15].

Previous studies demonstrated that the expression of miRNA-326 increased when the platelets were stored in vitro. Bioinformatic analysis showed that it targets the 3′-untranslated region (UTR) of Bcl-xL gene rendering it under its posttranscriptional regulation [8].

In this context, the aim of this study was to determine the differential expression level of miRNA-326 and its target genes (Bcl-xL and Bak) in stored platelet in blood banks at different points of storage, in order to highlight their role as a quality measuring tools for PSL.

## Materials and Methods

The current study was conducted on fifty PC bags (RDP) after platelet filtration using BioP plus leucocyte depletion filters (Fresenius Kabi, Hongkong), obtained from Alexandria Regional Blood Transfusion Center, during the period between September 2019 and March 2020. The study was conducted after approval of the Medical Ethics Committee of Alexandria Faculty of Medicine.

### Sampling from Platelet Bag

The PC bag was hanged vertically. The tube stripper was used to strip the contents of the tube into the bag. The platelet bag was then gently mixed for about 5 s. While keeping the stripper closed, the bag was vertically hanged again [16]. The stripper was then slowly moved up, allowing for slowly filling of the tube. The tube was heat sealed using a tube sealer. The tube was heat sealed again 1–2 inches above the previous seal to create the testing segment. Then the testing segment was cut off from the platelet unit for testing for day 0 [16].

Platelet bags were then placed on platelet agitator at 22 °C for 3 days, then previous steps to be repeated to be used for day 3 sample. Platelet bags were again placed on platelet agitator at 22 °C for 2 more days to be used for day 5 sample [16].

### Total RNA Extraction

Total RNA, including miRNA, isolation and purification from PC samples was carried out with the miRNeasy Mini Kit (QIAGEN, Maryland, USA, Cat No. 217004) according to the manufacturer's instructions. The concentration and purity of RNA were measured at 260, 280 and 230 nm using NanoDrop2000 Spectrophotometer (Thermo Scientific, USA). A260:A230 ratio greater than 1.7 and A260:A280 ratio greater than 2.0 indicates highly pure RNA.

### MiRNA-326 Relative Quantitative Expression

Single-stranded cDNA was synthesized from purified RNA samples by the TaqMan miRNA RT Kit (Applied biosystems, California, USA) according to the manufacturer instructions using SimpliAmp thermal cycler (Applied biosystems, USA).

Relative expression levels of miRNA-326 and the endogenous reference (RNU6B) were carried out by QR-PCR using the ready-made assays (ThermoFisher scientific, USA, Cat No. 4427975) [17].

The QR-PCR mixture contained 10 µl TaqMan universal master mix, 1 µl primers and probes, 7 µl RNase-free water and 2 µl cDNA in a total reaction volume 20 µl.PCR was done under the following conditions: 95 °C for 10 min followed by 40 cycles of 95 °C for 15 s and 60 °C for 1 min on Rotor Gene real time PCR system (Qiagen, USA).

### Relative Quantitative Expression of Bcl-xL and Bak

The cDNA was synthesized using High Capacity cDNA Reverse Transcription Kit (Applied Biosystems, USA). Briefly, 10 µl of RNA was reverse transcribed in to cDNA in a 20 µl reaction using random hexamer primers for 10 min at 25 °C, 120 min at 37 °C and 5 min at 85 °C using SimpliAmp (Applied biosystems, USA).

Expression levels of Bcl-xL and Bak genes were determined using sequence specific primers and relative to GAPDH, as an endogenous reference gene [18].

Used Primers sequences were as follows: **Bcl-xL forward**: TTACCTGAATGACCACCTA, **Bcl-xL reverse:** ATTTCCGACTGAAGAGTGA, **Bak forward**: GCCTACTGACCCAGAGATGG, **Bak reverse:** CTCATAGGCGTTGTCTGCTG, **GAPDH forward**: CGACTTCAACAGCGACACTCAC and **GAPDH reverse**:CCCTGTTGCTGTAGCCAAATTC. Determination of relative quantitative expression of Bcl-xL and Bak genes was carried out using QR-PCR on the Rotor Gene real time PCR system (Qiagen, USA).

The PCR mixture contained 12.5 µL Maxima SYBR®Green PCR master mix, 1 µl forward primers, 1 µl reverse primers, 8µL RNase-free water and 2.5 µl cDNA in a total reaction volume 25 µl. PCR was done under the following conditions: 95 °C for 10 min followed by 40 cycles of 95 °C for 30 s, 60 °C for 30 s and finally 72 °C for 1 min. followed by melting curve programmed as follows: 95 °C for 30 s followed by 55 °C for 30 s and finally 95 °C for 30 s.

### Data Analysis

Relative quantitation was expressed by a comparative Ct method where the amount of target, normalized to an endogenous reference and relative to a calibrator, was given by: 2^−∆∆Ct^ [19].

### Statistical Analysis

Statistical analysis was carried out using IBM SPSS software package version 20.0***.* (**Armonk, NY: IBM Corp**).**Quantitative data were tested for normality using Kolmogorov–Smirnov test.

Quantitative data were described using range (minimum and maximum), mean, standard deviation, median and inter quartile range (IQR).

Anova test was done for normally distributed quantitative variables, to compare between more than two periods or stages, and Post Hoc test (**Bonferroni adjusted**) was done for pair wise comparisons. For abnormally distributed quantitative variables, **Friedman test** was done to compare between more than two periods or stages and **Post Hoc Test** (**Dunn**'s**)** as done for pair wise comparisons. Pearson’s correlation was used for testing correlations between quantitative variables. Statistical significance was accepted as *p* < 0.05.

## Results

### Differential Expression Level of miRNA-326

There was significant over expression of miRNA-326 level along days of storage in blood bank with a mean expression level 1.04 ± 0.30 at **day 0**, 3.04 ± 1.05 at **day 3** and 6.34 ± 1.49 at **day 5** (*p* < 0.001). (Table 1).

**Table 1:** Relative quantitative expression of miRNA-326, Bcl-xL and Bak gene at different points of storage in blood bank

	Day 0	Day 3	Day 5	Test of Sig	*p*
*miRNA-326*					
Mean ± SD	1.04^c^ ± 0.30	3.04^b^ ± 1.05	6.34^a^ ± 1.49	F = 277.508^*^	< 0.001^*^
Median (Min. – Max.)	0.94 (0.52 – 1.74)	3.02 (1.77 – 7.06)	6.26 (3.89 – 9.71)		
Sig. bet. periods	*p*_*1*_ < 0.001^*^, *p*_*2*_ < 0.001^*^, *p*_*3*_ < 0.001^*^				
*Bak*					
Mean ± SD	1.15 ± 0.66	4.37 ± 2.52	7.10 ± 4.09	Fr = 100.0^*^	< 0.001^*^
Median (Min. – Max.)	0.99^c^ (0.37 – 4.02)	3.77^b^ (1.41 – 15.32)	6.12^a^ (2.29 – 24.89)		
Sig. bet. periods	*p*_*1*_ < 0.001^*^, *p*_*2*_ < 0.001^*^, *p*_*3*_ < 0.001^*^				
*Bcl-xL*					
Mean ± SD	1.092^a^ ± 0.432	0.180^b^ ± 0.071	0.011^c^ ± 0.004	F = 282.468^*^	< 0.001^*^
Median (Min. – Max.)	1.004 (0.127–2.282)	0.166 (0.021– 0.376)	0.010 (0.001– 0.024)		
Sig. bet. periods	*p*_*1*_ < 0.001^*^, *p*_*2*_ < 0.001^*^, *p*_*3*_ < 0.001^*^				

**F: F test (ANOVA) with repeated measures,** Sig. between periods was done using **Post Hoc Test** (**adjusted Bonferroni)**

**Fr**: **Friedman test**, Sig. between periods was done using **Post Hoc Test** (**Dunn's)**

*p*: *p* value for comparing between the studied periods

p_1_: *p* value for comparing between **Day 0** and **Day 3**

p_2_: *p* value for comparing between **Day 0** and **Day 5**

p_3_: *p* value for comparing between **Day 3** and **Day 5**

*Statistically significant at *p* ≤ 0.05

Means /Medians with Different letters are significant

### Differential Expression Level of Bak Gene

There was significant over expression of Bak gene level along days of storage in blood bank with a median expression level of 0.99 (0.37 – 4.02) at **day 0**, 3.77 (1.41 – 15.32) at **day 3** and 6.12 (2.29 – 24.89) at **day 5** (*p* < 0.001). (Table 1).

### Differential Expression Level of Bcl-xL Gene

There was significant decreased expression levels of of Bcl-xL gene along days of storage in blood bank with a mean expression level of 1.092 ± 0.432 at **day 0**, 0.180 ± 0.071 at **day 3** and 0.011 ± 0.004 at **day 5** (*p* < 0.001). (Table 1).

### Correlation Between miRNA-326, Bcl-xL and Bak Levels

**Fig. 1:**
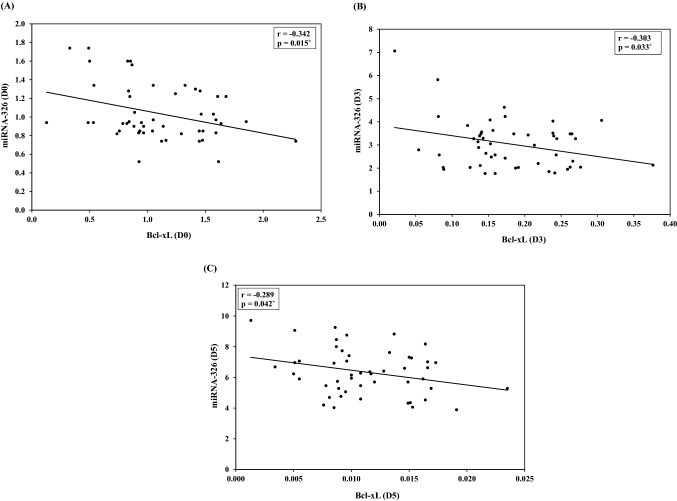
Correlation between miRNA-326 and Bcl-xL

A statistically significant negative correlation between miRNA-326 and Bcl-xL gene expression levels at **day 0** (r =  − 0.342, *p* = 0.015), **Day 3** (r =  − 0.303, *p* = 0.033) and **Day 5** (r =  − 0.289, *p* = 0.042) was reported (Fig. 1, Table 2).

**Table 2:** Correlation between miRNA-326, Bak and Bcl-xL gene expression at different points of storage in blood banks

	Day 0		Day 3		Day 5	
	r	*p*	r	*p*	r	*p*
miRNA-326vs.Bak	0.308	0.030^*^	0.428	0.002^*^	0.351	0.012^*^
miRNA-326vs.Bcl-xL	− 0.342	0.015^*^	− 0.303	0.033^*^	− 0.289	0.042^*^
Bak vs. Bcl-xL	− 0.021	0.887	− 0.170	0.237	− 0.078	0.589

**r: Pearson coefficient**

*Statistically significant at *p* ≤ 0.05

**Fig. 2:**
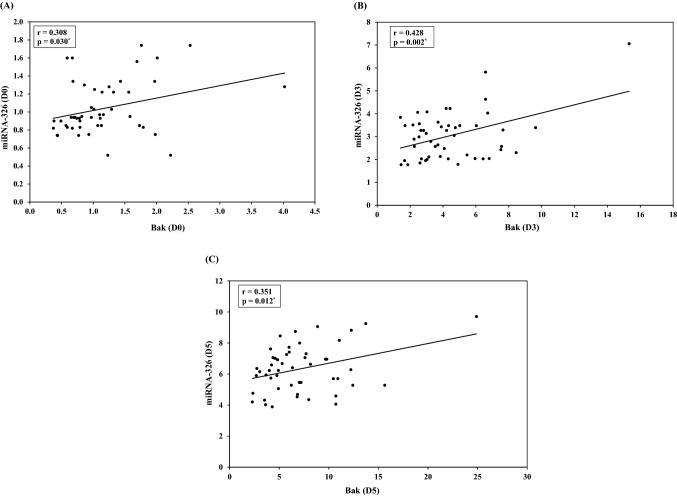
Correlation between miRNA-326 and Bak

Also, a statistical significant positive correlation was observed between miRNA-326 and Bak expression levels at **day 0** (r = 0.308, *p* = 0.030), **Day 3** (r = 0.428, *p* = 0.002) and **Day 5** (r = 0.351, *p* = 0.012) (Fig. 2, Table 2).

No statistically significant correlation could be detected between Bak and Bcl-xL genes expression levels at **day 0** (r =  − 0.021, *p* = 0.887), **day 3** (r =  − 0.170, *p* = 0.237) or **day 5**(r =  − 0.078, *p* = 0.589) (Table 2).

## Discussion

The continuous raise in the demand of platelet transfusion has necessitated the need to establish novel standards for determining the quality of PC units during storage. In the last few years, early and precise detection of the PSL becomes a subject of great interest in transfusion practice. Extensive research have been implemented to distinguish PC bags underwent PSL, from those with functional platelets. Recently, several researches were carried out to highlight the role of platelets' miRNAs as a platelet efficiency quality measuring tool for PCs bags stored in blood bank [20].

MiRNA differential expression profile was found to be a helpful tool for recognition of the molecular background of the physiological platelet changes occurring during storage [5, 14, 20].

**The current study aimed to** highlight the role of platelet **miRNA-326** and its putative target apoptotic genes, Bcl-xL and Bak, as key players in apoptosis regulation in stored PC units resulting in PSL over days of platelet storage in blood bank. MiRNA-326, Bcl-xL and Bak expression levels were measured by QR-PCR at day 0, day 3 and day 5 of storage in blood bank.

The results of this study showed significant upregulation in miRNA-326 over days of storage in blood bank with increasing levels from day 0 to day 3 then day 5.

Since platelets are anucleated, they are unable to synthesize new miRNAs. Yet, increased expression of miRNAs might be due to their synthesis from pre-miRNAs already present in platelets through cleavage by RNA editing enzymes. This theory was affirmed by detection of both pre-miRNA transcripts and the components of the miRNA effector complex within platelets [9].

Similar results to ours were reported by Yu et al. [8] who used QR-PCR on apheresis platelets to analyze platelet apoptosis associated miRNAs at baseline (day 0) and after 1, 3 and 5 days of storage. Yu et al. found significant up-regulation of a panel of miRNAs including in miRNA-326 expression levels throughout storage days. These findings suggested a role for miRNAs in regulating platelet physiology in ex vivo conditions [8].

Platelets life span is regulated by Bcl-2 family proteins which balance the initiation or inhibition of apoptosis. Bcl-2 family members includes anti-apoptotic,e.g. Bcl-2, Bcl-xL and Mcl-1and pro-apoptotic, e.g.Bax and Bak [21]. Pro-apoptotic proteins interact with mitochondrial outer membrane. This leads to mitchondrial outer membrane permeabilization (MOMP) and release of cytochrome c that triggers the apoptotic cascade. On contrary, anti-apoptotic proteins, like Bcl-xL, maintain cellular viability probably through restraining pro-apoptotic proteins Bax and Bak or both of them [22].

Because platelets lack a functional nucleus, the regulation of mRNAs cannot be done by DNA transcription or replication. Consequently, the regulation of the apoptosis genes in platelets is mostly post-transcriptional. Several researches have confirmed that platelets contain a large repertoire of miRNAs that regulate platelet gene expression profile.Furthermore, previous studies showed that both Bcl-xL and Bak are putative target genes for miRNA-326. [22]

In the present study Bcl-xL showed proggressive downregulation in PCs from day to day during storage in blood bank. While Bak showed significant upregulation from day 0 to day 3 then day 5. A significant negative correlation between miRNA-326 and Bcl-xL and a positive one with Bak.

This data is supported by the findings of Mason et al. [23] who found that older platelet contain less Bcl-xl than younger platelets. They also found that a dose-dependent reduction of platelet survival and life span takes place when Bcl-xL genetic mutation or pharmacological antagonism occurs, unlike other pro-apoptotic proteins which play lesser roles [23]. In this study when genetic ablation of Bcl-xL was done, it lead to reduction in platelet half life and subsequently thrombocytopenia. On the other hand, platelets from Bak-deficient mice have longer life span than normal;when simultaneous deletion of Bak took place.That’s why they emphasized on the role of Bcl-xL and Bak in platelets apoptosis considered them the chief proteins that affect storage-related platelet apoptosis [23, 24].

In the same direction, Yu et al. [22] found that miRNA-326 reduces not only Bcl-xL mRNA but also its protein levels. They found that when miRNA-326 expression inhibition was done, there was an increase in Bcl-xL mRNA and protein expression levels by using dual luciferase reporter assay. They found that after miRNA-326 transfection there was a major rise in the apoptotic activity in platelets [22].

Similarily, Qiao et al. [25] who used dual luciferase reporter gene vectors for Bcl-xL -3'UTR-WT (wild-type) and Bcl-xL -3' UTR-MT (variant), found that miRNA-326 regulates platelet life span by acting on the 3'-UTR of the Bcl-xL gene [25].

MiRNA-326 regulates Bcl-xL expression through direct binding with complete complementarity to the 3’-UTR region of Bcl-xL mRNA.The positive correlation between miRNA-326 and Bak gene expression levels may probably be through an indirect mechanism as mentioned by Yu et al. [22], through inhibition of Bcl-xL expression by miRNA-326 and so enhancing Bak expression and inducing apoptosis in stored platelets [22].

The insignificant correlation between Bcl-xL and Bak may be explained by the observation of Yan et al. [24], that anti apoptotic Bcl-xL is degraded more rapidly than Bak over time, both in vivo and in vitro [24]. Similarily, Mason et al. [23] who found that Bcl-xL and Bak have different half lives [23].

However, some limitations of the current study should be acknowledged. Due to financial limitations, we carried out the current study in a small sample size and we did not study the criteria of each PC unit individually using automated cell analyzer. Also, no correlation of miRNA levels with either the platelet content or the WBC content of the unit had been done. Moreover, larger multicenter studies to confirm our findings are warranted, as well as to investigate the possible molecular networks regulating platelet apoptosis and PSL and their clinical efficiency in transfused patients.

## Conclusion

From the current study results we can conclude that,MiRNA-326 is overexpressed along time during platelet storage and may promote platelet apoptosis and PSL through inhibition of anti-apoptotic Bcl-xL gene expression and enhancing pro-apoptotic Bak expression. This highlighted the clinical applicability of miRNA-326 as a useful tool and potential quality biomarkers of platelets viability during storage in blood banks which may provide insights for enhancing platelets quality and adjusting shelf life.

## Data Availability

All data and raw material are available upon request.
